# *CCL2* Gene Expression and Protein Level Changes Observed in Response to Wingate Anaerobic Test in High-Trained Athletes and Non-Trained Controls

**DOI:** 10.3390/ijerph19169947

**Published:** 2022-08-12

**Authors:** Agnieszka Maciejewska-Skrendo, Maciej Tarnowski, Patrycja Kopytko, Andrzej Kochanowicz, Jan Mieszkowski, Błażej Stankiewicz, Marek Sawczuk

**Affiliations:** 1 Faculty of Physical Culture, Gdansk University of Physical Education and Sport, 80-336 Gdansk, Poland or; 2 Institute of Physical Culture Sciences, University of Szczecin, 71-065 Szczecin, Poland or; 3 Department of Physiology, Pomeranian Medical University, 70-111 Szczecin, Poland; 4 Institute of Physical Culture, Kazimierz Wielki University, 85-091 Bydgoszcz, Poland

**Keywords:** CCL2, chemokine, gene expression, athletes, training adaptation, inflammatory response

## Abstract

Intensive, acute exercise may bring a large systemic inflammatory response marked by substantial increases in inflammatory cytokines and chemokines. One such chemokines–CCL2–is a key factor involved in inflammatory reaction to exercise. The direct aim of the study was to describe the changes in the *CCL2* expression levels after anaerobic exercise in well-trained athletes adapted to long-term training and in non-trained participants. The expression of *CCL2* mRNA was evaluated in peripheral blood MNCs and CCL2 protein level was observed in blood plasma. The changes were assessed as the response to an acute, intensive bout of exercise (Wingate Anaerobic Test) in two groups of participants: well-trained soccer players and non-trained individuals. An increase of *CCL2* expression inn both mRNA and protein levels was observed. The response was greater in non-trained individuals and elevated levels of *CCL2* transcripts persisted for more than 24 h after exercise. Well-trained individuals responded more modestly and the effect was attenuated relatively quickly. This shows muscular adaptation to a continuous training regime in well-trained individuals and better control of immune reactions to muscular injury. In non-training individuals, the induction of the inflammatory response was greater, suggesting presence of more serious myotrauma.

## 1. Introduction

It is well known that physical activity (PA) reduces the risk of chronic diseases such as cardiovascular disease, type 2 diabetes, and some types of cancers [1,2,3,4,5,6,7]. PA is also greatly important in regulating energy demand and expenditure, thus enabling proper whole-body glucose homeostasis. The body’s response to physical exercise persists on many levels: molecular, cellular, tissue, organ, and systemic. The response is dependent, among others, on type of physical exercise, type of training, and the degree of training.

Intensive, acute exercise, commonly of anaerobic character, may bring some certain negative effects like significant tissue stress, injury, and a large systemic inflammatory response marked by substantial increases in several inflammatory cytokines and chemokines [8,9,10,11,12,13,14], known as an acute-phase response (APR). If the inflammation is not resolved, with little recovery time, it may turn to chronic low-grade inflammation. The APR is triggered by acute anaerobic exercise and is characterized by the release of multiple biologically active substances that may be of pro- or/and anti-inflammatory character. It is known that acute vigorous exercise has a great impact on almost all immune cell populations [15,16]. The most prominent effect is triggered in muscles and blood cells, and there is a significant interplay between these two tissues that on one side leads to systemic inflammation and on the other is necessary for muscular regeneration, recovery, further resolution of the inflammation, and adaptation of the immune response [12,13]. Mechanistically, acute exercise may be characterized by the activation of the innate immune system by tissue damage, hemodynamic stimuli such as sheer stress, blood flow, or oxidative stress, leading to the release of chemokines and infiltration of immune cells into injured areas such as skeletal muscles [9,10,11,12]. The molecules released by intensively exercising muscle are termed myokines, and in a broader perspective, the molecules that are active and secreted in response to exercise are termed exerkines or exercise factors [8,12,17,18]. Well-characterized exerkines include interleukin 1-beta (IL-1B), interleukin 6 (IL-6), and tumor necrosis factor alpha (TNF-a); these are known pro-inflammatory cytokines and are released in the early stages of inflammation [17,19]. Interleukin 10 (IL-10) is an anti-inflammatory cytokine released later to repress the pro-inflammatory effects and to support the potential differentiation of muscle stem cells [19]. Recent research shows great involvement in both regeneration of muscle tissue and inflammation of specific chemokines such as monocyte chemoattractant protein-1 [MCP-1, chemokine nomenclature: C–C motif chemokine ligand 2 (CCL2)] [20]. 

CCL2 is a ligand of G-protein-coupled receptor C–C chemokine receptor type 2 (CCR2) [21]. *CCL2* is expressed by great number of cells, such as leukocytes, myeloid cells, endothelial cells, muscle cells, fibroblasts, epithelial cells, and tumor cells (reviewed in [20]). *CCL2* expression is stimulated by exposure to inflammatory inducers, growth factors such as platelet-derived growth factor (PDGF), vascular endothelial growth factor (VEGF), or hypoxia [20,21,22,23,24]. *CCL2* mRNA expression is dramatically upregulated in injured tissues; the chemokine is released to circulation and triggers chemotaxis, extravasation, and the migration of macrophages to the sites of regeneration and repair [25]. Moreover, CCL2 activity is not only limited to the regulation of migration, it also triggers a vast array of inflammatory reactions, including the release of other chemokines and cytokines [20,26]. Since CCL2 is responsible for sustaining cellular recruitment, its prolonged or continuous release may induce pathogenic, chronic inflammatory reactions as the one seen in multiple sclerosis [27], atherosclerosis [28], and rheumatoid arthritis [29]. What is also important is that weight loss and regular exercise lead to a reduction in CCL2 levels and improved insulin sensitivity [30,31,32].

Even though its role seems to be of key importance in regulating inflammatory processes and cellular reaction to exercise, the results obtained by researchers differ. For this reason, we decided to study the effect of very intense effort on the expression of the *CCL2* gene, which encodes this chemokine. We assumed that the effect of intense anaerobic exercise would be different in individuals permanently adapted to such physiological stress conditions by years of athletic training than in physically inactive individuals. Therefore, professional athletes with many years of regular sports training were recruited for the study group. The direct aim of the study was to describe the changes in the *CCL2* expression levels after a single bout of high intensity, supramaximal anaerobic exercise in well-trained elite athletes adapted to long-term sports training and in non-trained participants.

## 2. Material and Methods

The study design is an interventional and analytical study aimed at testing the hypothesis that the magnitude and/or direction of changes in *CCL2* gene expression observed under the influence of supramaximal anaerobic exercise are different in trained and physically inactive subjects. An experiment based on an ex post facto research design was used for the study because the independent variable (group variable) could not be manipulated. Throughout the experiment, the group (“Trained”, “Non-trained”) was the independent variable, whereas the expression levels of the *CCL2* gene measured in pre-test and post-tests were the dependent variables. Recruitment of the “Trained” (study group) and “Non-trained” (control group) participants followed the principle of purposive selection.

### 2.1. Participants

The procedures used in the study were conducted ethically according to the principles of the Declaration of Helsinki of the World Medical Association and ethical standards in sports and exercise science research. The entire experimental protocol was approved by the Ethics Committee of the Regional Medical Chamber in Gdansk (approval number: KB-35/17). All participants received an informed consent form and a written information sheet about the study with all relevant information (purpose, procedures, risks, benefits of participation). All participants were given time to read the information sheet and consent form. After making sure that they understood the information, all participants agreed in writing to participate in the project (signed informed consent), making sure that the project was anonymous and that the results obtained would be kept confidential. The experimental procedures were planned in accordance with the principles for reporting the results of genetic association studies as set forth in the STrengthening the REporting of Genetic Association Studies (STREGA) Statement [33].

Participants for the study group (referred to as “Trained”) were recruited through direct contact with coaches, by targeting national teams, and by providing information to athletes attending training camps and sport trials, as well as through public announcements or voluntary letters of intent. The criteria for inclusion in the “Trained” group were:

- gender (males only),
- all participants of the study group were qualified in the lists of Polish sports federations for their discipline, which means that they had a licence to compete,
- all participants had a documented history of systematic sports training and participation in a specific sport for over 10 years,
- all participants had regular medical examinations to allow them to participate in sports and compete,
- good current health status (no concurrent injuries) and a negative medical interview for cardiovascular system disorders, autonomic nervous system disorders, mental disorders, head injuries, and other diseases that could directly affect the experimental procedures,
- no intake of dietary supplements or medications during the study that could directly affect the results obtained.

To achieve the objectives of the study, 20 experienced soccer players (mean age 22.5 ± 5.3 years) were recruited for the “Trained” group.

Non-trained men were recruited for the control group (referred to as “Non-trained”) on the basis of a public announcement or voluntary letters of intent. The criteria for inclusion in the “Non-trained” group were:

- gender (males only),
- control participants were identical to those in the study group in terms of age and characteristics of general morphological indicators,
- a low level of physical activity, self-reported by each participant using the Global Physical Activity Questionnaire (according to the World Health Organisation Polish version),
- a good current state of health (no concurrent injuries) and negative medical questioning about disorders of the cardiovascular system, autonomic nervous system, mental disorders, head injuries, and other diseases that could directly affect the experimental procedures,
- no intake of dietary supplements or medications during the study that could directly affect the results obtained.

To achieve the objectives of the study, 20 participants (mean age 20.9 ± 1.1) were recruited for the “Non-trained” group.

### 2.2. Measurement of Anaerobic Capacity by the Wingate Anaerobic Test

To evaluate the effects of supramaximal anaerobic effort on the changes in *CCL2* expression levels, anaerobic capacity measurements were performed using the Wingate Anaerobic Test (WAnT) in the 30-s version with full resistance of the flywheel from the beginning of the effort, as it was described previously [34]. All participants performed the lower limb test on an Ergomedic E818 two-wheeled ergometer (Monark, Vansbro, Sweden) with individually selected flywheel resistance (0.075 kg per kg body weight). The computer program MCE v5.1 was used to calculate the mechanical parameters in this test. The following parameters were calculated: total work (Wtot), maximum anaerobic power (PPWAnT), time to reach maximum power (TUZ), time to maintain maximum power (TUT) to the nearest 0.01 s, and power decrease (WSM) in percentage values.

### 2.3. Biological Material Samples Collection

Venous blood samples (volume 500 μL) were collected from the “Trained” and “Non-trained” participants before WAnT (pre-test) and immediately after WAnT (0 h) and 30 min (0.5 h), 6 h (6 h) and 24 h (24 h) after WAnT (post-tests) in the RNAprotect^®^ Animal Blood Tubes (Qiagen, Hilden, Germany). In addition, blood plasma was collected for ELISA (Enzyme-linked Immunoassay) evaluation.

### 2.4. Isolation of the RNA and cDNA Synthesis

Total RNA was extracted from blood samples using an RNeasy^®^ Protect Animal Blood Kit (Qiagen, Hilden, Germany) according to the manufacturer’s protocol. All extracts were treated with DNAse to avoid contamination with genomic DNA. The obtained isolates were spectrophotometrically tested for RNA quantity and quality using a spectrophotometer (Eppendorf, Hamburg, Germany). A total of 100 ng of each RNA sample was subjected to reverse transcription using the iScript cDNA Synthesis Kit (Biorad, Hercules, CA, USA), and the resulting cDNA samples were stored at −20 °C until further analysis.

### 2.5. Application of Quantitative Real-Time PCR

To evaluate the expression of the *CCL2* gene, a Quantitative Real-Time PCR (QRT-PCR) was performed in duplicate for each sample using a StepOne Real-Time Polymerase Chain Reaction instrument (Applied Biosystems, Foster City, CA, USA). QRT-PCR fluorescence detection was performed in 96-well low-skirted plates (Applied Biosystems, Foster City, CA, USA) using a PrimePCR™ SYBR^®^ Green Assay (Biorad, Hercules, CA, USA) and SsoAdvanced Universal SYBR Green SuperMix Reagents Kit (Biorad, Hercules, CA, USA) according to the manufacturer’s protocol under the following conditions: 95 °C for 30 s for initial denaturation, followed by 40 cycles of 10 s at 95 °C for denaturation and 30 s at 60 °C for annealing, extension, and plate reading. A quantified transcript of the gene encoding human glyceraldehyde-3-phosphate dehydrogenase (*GAPDH*) was used as a housekeeping gene, and no significant differences in *GAPDH* mRNA copy number were detected between the analyzed groups.

### 2.6. Enzyme-Linked Immunoassay

Blood plasma was collected from all study participants at the indicated time points. Plasma levels of CCL2 were determined using a CCL2 enzyme immunoassay (R&D Systems, Minneapolis, MN, USA) according to the manufacturer’s instructions.

### 2.7. Statistical Analysis

Preliminary statistical analyses included calculation of mean values and standard deviations of gene expression parameters and analysis of *CCL2* gene expression levels obtained from the QRT-PCR reaction using relative quantification of a target gene compared to a reference gene method [35]. The expression ratio of the *CCL2* gene normalized with the expression of the *GAPDH* reference gene measured for the samples in the “Trained” and “Non-trained” groups before WAnT was used as a calibrator (2^−^^ΔΔCt^ = 1).

With regard to CCL2 protein levels, calculation of means and standard deviations and analysis of direct CCL2 protein levels observed in enzyme immunoassay tests were used.

The distribution of variables was assessed with the Shapiro-Wilk test. Because most of the data were not normally distributed, the post hoc nonparametric Mann-Whitney U-test was used to assess significant differences between *CCL2* gene expression levels as well as CCL2 protein levels observed in and between the “Non-trained” and “Trained” groups at post-test time points (0 h, 0.5 h, 6 h, 24 h). To examine the correlation between the level of *CCL2* gene expression observed in a given post-test and the amount of CCL2 protein produced, Spearman and Kendall rank correlation analysis were performed.

Results were considered statistically significant for *p* < 0.05. All calculations were performed using Microsoft Excel 2017 (Microsoft, Redmond, WA, USA) and PQStat software (PQStat Software, Poznań, Poland).

## 3. Results

**Figure 1:**
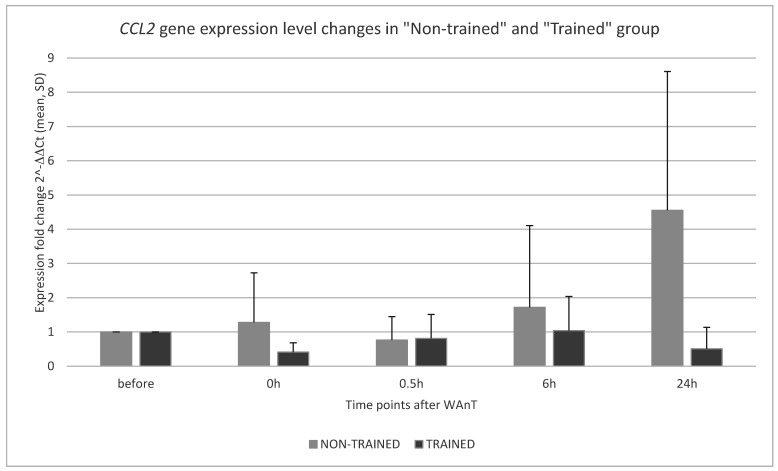
*CCL2* gene expression level changes in “Non-trained” and “Trained” group in four post-tests after WAnT. (The expression fold change measured before WAnT test was used as calibrator (2^−^^ΔΔCt^ = 1).)

The results of *CCL2* expression changes calculated by the ΔΔCT method between the “Non-trained” and “Trained” groups at four time points after WAnT testing are shown in Figure 1.

**Figure 2:**
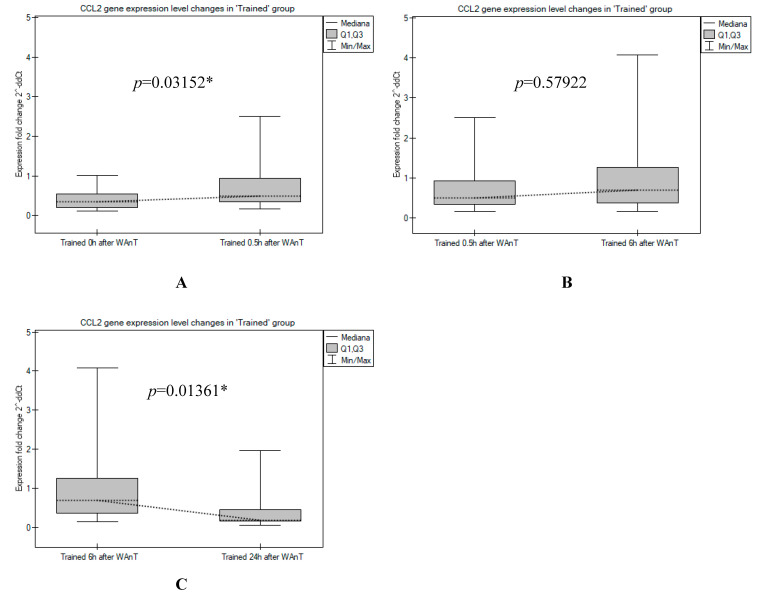
A comparison between the *CCL2* gene expression levels measured in the “Trained” group in post-tests: (**A**) 0 h vs. 0.5 h after WAnT; (**B**) 0.5 h vs. 6 h after WAnT; and (**C**) 6 h vs. 24 h after WAnT. (*p* values are Mann–Whitney U-test asymptotic *p* values corrected for ties, * statistically significant differences).

In the “Trained” group, *CCL2* mRNA expression showed a minimal level when measured immediately after the test, followed by a continuous increase over time with a maximum at the sixth hour after WAnT, whereas *CCL2* transcript levels reached a level comparable to that observed immediately after WAnT at 24 h (Figure 1). The increase in *CCL2* transcripts measured between time points 0 h and 0.5 h and the rapid decrease between time points 6 h and 24 h were found to be statistically significant (*p* = 0.03152 and *p* = 0.01361, respectively; Figure 2).

**Figure 3:**
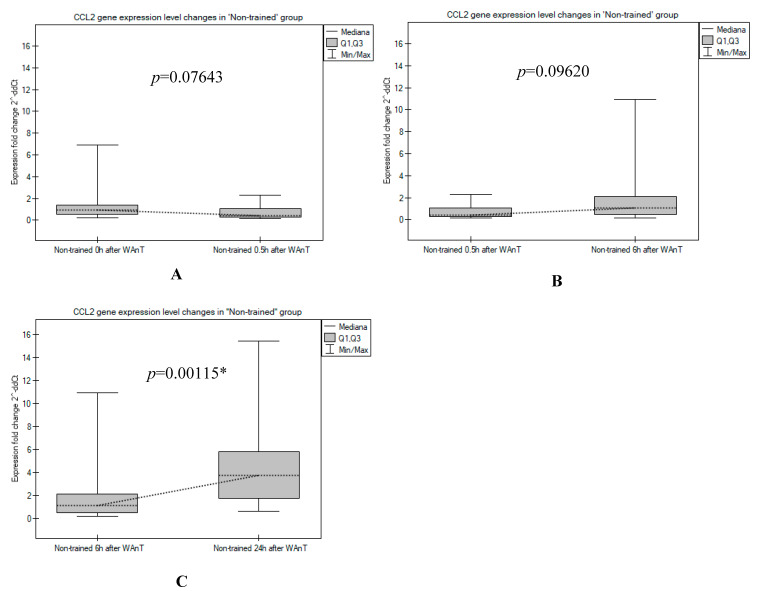
A comparison between the *CCL2* gene expression levels measured in the “Non-trained” group in post-tests: (**A**) 0 h vs. 0.5 h after WAnT; (**B**) 0.5 h vs. 6 h after WAnT; and (**C**) 6 h vs. 24 h after WAnT (*p* values are Mann–Whitney U-test asymptotic *p* values corrected for ties, * statistically significant differences).

The trend of changes in *CCL2* expression level observed in the “Non-trained” participants was clearly different: the number of *CCL2* transcripts was relatively high in the first period after the WAnT test (time point 0 h), then the observed *CCL2* expression dropped to a lower level (0.5 h after WAnT). A continuous increase in *CCL2* transcripts was recorded at the next two time points, with a maximum observed 24 h after WAnT (Figure 1). Indeed, the increase in *CCL2* transcripts between time points 6 and 24 h was statistically significant (*p* = 0.00115; Figure 3).

A direct comparison of measured *CCL2* expression levels between the “Non-trained” and “Trained” groups in four post-tests after WAnT revealed that *CCL2* transcript levels measured immediately after WAnT (time point 0 h) and 6 h and 24 h after WAnT were significantly higher in the non-trained participants than in the trained soccer players. Only at the time point 0.5 h after WAnT, the expression of *CCL2* was almost the same in the “Trained” and “Non-trained groups” (Figure 1). Statistical comparisons between *CCL2* expression levels measured in the “Non-trained” and “Trained” groups during subsequent post-tests showed that the observed differences at time points 0 h and 24 h after WAnT were statistically significant (*p* = 0.00034 and *p* = 0.00002, respectively).

**Figure 4:**
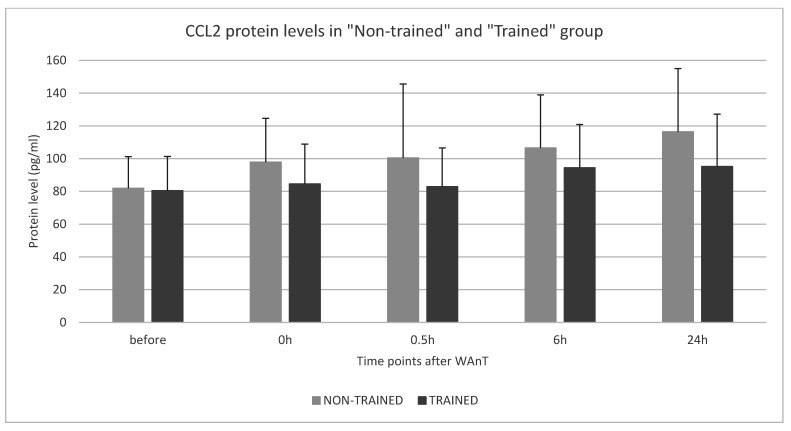
CCL2 protein level changes in “Non-trained” and “Trained” group in four post-tests after WAnT.

The results of the measurements of the CCL2 protein levels between the “Non-trained” and the “Trained” group in the pre-test and at four time points of the post-tests after WAnT are shown in Figure 4. 

In the “Non-trained” group, the amount of CCL2 protein increased slowly but steadily from the beginning. In the “Trained” group, a slight decrease in CCL2 protein was measured between time points 0 h and 0.5 h followed by a slow increase until the last time point. However, all observed changes in CCL2 protein levels were found to be statistically insignificant in both groups: “Non-trained” and “Trained”.

A direct comparison of measured CCL2 protein levels between the “Non-trained” and “Trained” groups in four post-tests after WAnT revealed that CCL2 levels were significantly higher in the non-trained participants compared to the trained players. However, all observed differences between the two groups (“Non-trained” vs. “Trained”) were found to not be statistically significant.

The results of correlation analyses performed for comparisons between *CCL2* gene expression level and CCL2 protein are shown in Table 1. The statistically significant positive correlations between direct *CCL2* expression levels (2^−^^ΔCt^ values) and CCL2 protein levels (protein concentrations) were observed only for the “Trained” group in two post-tests: 0 h (*p* = 0.017131 and *p* = 0.044268, respectively, for Spearman’s and Kendall’s rank correlation) and 0.5 h (*p* = 0.022161 and *p* = 0.027518, respectively, for Spearman’s and Kendall’s rank correlation). All other correlations (both positive and negative) observed in the “Trained” and “Non-trained” groups were estimated to be statistically insignificant.

## 4. Discussion

Acute exercise induces a significant systemic inflammatory response named APR, marked by substantial increases in several inflammatory cytokines and chemokines [36,37,38]. The APR includes a complex mediator cascade aimed towards minimizing the expansion of tissue damage, enabling recovery from proinflammatory processes i.e., transiently redistributing immune cells to peripheral tissues, resulting in a proper immune regulation, regeneration, and tissue repair [39]. APR in its timing should be transient and limited, as it is known that a persistent inflammatory response is associated with tissue dysfunction and pathology [40].

In the study, we evaluated the expression of *CCL2* mRNA in peripheral blood MNCs and protein level of CCL2 in blood plasma. The changes of CCL2 levels were assessed as the response to acute, intensive bouts of exercise-Wingate Anaerobic Test. The response was measured and compared in two groups of participants: well-trained experienced soccer players and non-training, low physical activity individuals. We noticed a significant increase in *CCL2* mRNA expression in peripheral blood mononuclear cells and protein levels of CCL2 after WAnT. A clear trend in the increase the expression of the *CCL2* gene after intense physical activity was observed only in the group of physically inactive individuals, whereas the level of *CCL2* gene transcripts observed after the test was much more balanced in the trained players. This could be the result of some adaptation at the molecular level to the physical exertion and repeated intense training that accompanies players for many years during their involvement in professional sports. This aspect will be discussed in more detail later in the discussion. It is also worth noting that the most significant increase in *CCL2* expression was detected 24 h after the test in the non-trained subjects. This suggests that observations of interest may relate to time points even later after exercise. This leads to the conclusion that additional time points (after 48 h and 72 h) should be considered in future studies of changes in expression of selected genes after intense exercise in subjects with different training statuses.

Although it has been shown that CCL2 is a highly responsive molecule to physical training, the number of studies showing a peripheral response to acute exercise in healthy men, in the context of CCL levels and MNCs expression, is somehow limited. However, numerous studies show increased local expression of CCL in muscle tissue after single or repeated bouts of exercise, both in animal models and in humans [11,41,42]. This response seems to be greatly important in triggering leukocytic (mostly monocytes and macrophages) mobility and migration, which seems necessary for proper muscle regeneration [20,25]. In contrast, there are studies showing a decrease of plasma levels pf CCL2 after exercise interventions in patients having undergone coronary artery bypass surgery [43], with metabolic syndrome [44] or suffering from obesity [31,45]. 

Studies performed on healthy subjects mostly show an increase in CCL2 levels on protein level in peripheral blood plasma/serum in various types of exercise regimens. CCL2 was shown to be increased in acute bout of high-intensity, high-volume lower-body resistance exercise [46], and marathon racing [47]. In the study by Catoire et al., CCL2 was identified as a myokine whose expression in muscle and plasma level increases after 1-h one-legged acute exercise [12]. What is more, increased plasma levels of CCL2 were found in female rowers after 1 h of endurance exercise [48] and in males subjected to 36 h fasting trial and a subsequent acute bout of moderate exercise [49]. Zwetsloot et al. noted that a single acute session of high-intensity interval training (HIIT) (60 s, at 100% VO_2_max) in active young men led to an increase in several inflammatory cytokines and chemokines, such as IL-6, IL-8, IL-10, TNF-α, and MCP-1 (CCL2) [50]. What is interesting is that the authors found no difference in inflammatory response after 2 weeks of training. These results are in agreement with the results obtained here, as in our study, well-trained individuals’ response was somehow lower than non-training young men. That may be understood as repeated exercise being important in immune system adaptation to the continuous physical training. The time interval of the rest after acute exercise has a very profound attenuating effect on the inflammatory response. As mentioned before, in our study we noted that *CCL2* expression in MNCs was reduced to an entry level in well-trained individuals but non-trained participants continued to have altered *CCL2* expression even after 24 h post-exercise. It is known that CCL2 plays an important role in activating monocytes in muscle regeneration after a damage to the tissue [20,25]. Hence, it may be speculated that in non-physically active individuals, the tissue stress is much greater after intensive exercise that involves stronger and more extensive pro-regenerative response directed by CCL2. CCL2 is known to be increasingly expressed with repeated bouts of exercise, suggesting that CCL2 plays a key role in muscle adaptation. In the recent study by Middelbeek et al., it is shown that sprint interval training did not alter CCL2 concentrations, but importantly, the moderate intensity training conducted within a two-week period increased MCP-1 concentrations by 14%. What is more, CCL2 level in blood plasma correlated with other cytokines (IL-8 and TNF-α), with glucose uptake and HDL [51].

On the mRNA level, it was shown that *CCL2* expression in PBMNCs is generally higher in older people, and it is ameliorated by training [52,53,54]. Gjevestad et al. have shown a significant increase in *CCL2* mRNA expression after high intensity exercise in muscle biopsies and no significant change in mRNA expression in PBMNC [55]. The results suggest a different mode and timing of response when muscle tissue was compared to PBMNCs. 

*CCL2* is expressed by numerous tissues, including muscles, fat tissue, and leukocytes. Appropriate muscular regeneration is maintained by the migration of monocytes and macrophages to the sites of injury [56,57,58]. Injured muscle secretes factors that are chemotactic for macrophages and other inflammatory cells [59], the essential cells in the removal of damaged or necrotic tissue [60]. Furthermore, macrophages enhance the proliferation and delay differentiation [61] of satellite cells, the multipotent myogenic stem cells that are integral to regenerating damaged muscle [62]. It was shown that in the absence of MCP-1 (MCP-1 knockout mice), alterations in neutrophil recruitment and tissue chemotactic factors occurred in parallel with impaired skeletal muscle regeneration. Given the available biological samples in our study, we wanted to check the existing correlations between the genetic expression (mRNA) of *CCL2* gene in MNCs and protein levels of CCL2 in peripheral blood plasma. We found that the correlation between protein concentration and expression level of the *CCL2* gene exceeded the threshold of statistical significance only in trained participants in the period immediately after the test. However, the values of Spearman’s r coefficient calculated for these correlations are relatively low (at the level of 0.5), i.e., at the limit of values assumed to be significant (in biological sciences, these are usually values in the range of 0.7 < r < −0.7 [63]). On this basis, we assumed that it is too weak a premise to consider these correlations as strong—we assumed that it is a limited correlation. Therefore, we consider that our results revealed the presence of a limited correlation between the genetic expression of *CCL2* gene in MNCs and protein levels of CCL2 in peripheral blood plasma, indicating that MNCs are not the main origin of CCL2 and that acute exercise affects multiple plausible sources of *CCL2* expression. Recent investigations indicated that MCP-1 may have important effects on the healing process beyond its mononuclear cell recruiting properties. MCP-1 has direct angiogenic effects, and human endothelial cells express the MCP-1 receptor CCR2 [64]. CCL2 seems to be involved in inflammation-driven angiogenesis and atherosclerosis [65,66]. Furthermore, MCP-1 may directly modulate fibroblast phenotype and activity by increasing collagen expression [67] and by modulating matrix metalloproteinase expression [68]. Hence, MCP-1 may exert diverse effects on different cell types involved in the postinfarction inflammatory response. 

## 5. Conclusions

In summary, we observed an increase in *CCL2* expression at both the mRNA and protein levels in peripheral blood and plasma MNCs, respectively. 

The most significant increase in *CCL2* expression was observed 24 h after exercise in the untrained subjects. The conclusion is that observations of interest may relate to time points even later after exercise, so additional time points (after 48 and 72 h) should be considered in future studies of changes in expression of selected genes after intensive exercise in subjects with different training statuses.

Well-trained subjects responded more modestly, and the effect was attenuated relatively quickly. This picture presumably demonstrates muscular adaptation to a continuous training regimen in well-trained subjects and a tighter control of immune responses to Wingate test-induced muscle injury. In untrained subjects, the induction of the inflammatory response is more extensive, suggesting the presence of more severe myotrauma.

## Figures and Tables

**Table 1:** The results of correlation analyses carried out for comparisons between the expression level of the *CCL2* gene and the CCL2 protein.

Group	Time Point	r_s_	*p* ^#^	τ	*p* ^^^
“Trained”	0 h after WAnT	0.526316	0.017131 *	0.326316	0.044268 *
	0.5 h after WAnT	0.521053	0.022161 *	0.368421	0.027518 *
	6 h after WAnT	0.242105	0.303755	0.126316	0.436178
	24 h after WAnT	−0.121429	0.666401	−0.12381	0.520008
“Non-trained”	0 h after WAnT	0.260641	0.281148	0.211145	0.206525
	0.5 h after WAnT	−0.138596	0.571491	−0.122807	0.462524
	6 h after WAnT	−0.312782	0.179364	−0.252632	0.119393
	24 h after WAnT	−0.283516	0.325966	−0.252747	0.207982

r_s_ Spearman’s rank correlation coefficient calculated for comparisons between the expression levels of the *CCL2* gene (presented as direct 2^−^^ΔCt^ values) and the CCL2 protein (presented as direct protein concentrations [pg/mL]); ^#^ two-sided *p* calculated for r_s_; τ Kendall’s rank correlation coefficient calculated for comparisons between the expression levels of the *CCL2* gene (presented as direct 2^−^^ΔCt^ values) and the CCL2 protein (presented as direct protein concentrations [pg/mL]); ^^^ two-sided *p* calculated for τ; * statistically significant differences.
